# PLD1 regulates adipogenic differentiation through mTOR - IRS-1 phosphorylation at serine 636/639

**DOI:** 10.1038/srep36968

**Published:** 2016-11-22

**Authors:** Hae-In Song, Mee-Sup Yoon

**Affiliations:** 1 Department of Molecular Medicine, School of Medicine, Gachon University, Incheon 406-840, Republic of Korea

## Abstract

Phospholipase D1 (PLD1) plays a known role in several differentiation processes, but its role in adipogenic differentiation remains unknown. In the present study, we identified PLD1 as a negative regulator of adipogenic differentiation. We showed that PLD activity was downregulated by both 3-Isobutyl-1-methylxanthine (IBMX) and insulin upon induction of differentiation in 3T3-L1 adipogenic cells. In line with this observation, PLD activity decreased in both high fat diet (HFD)-fed mice and ob/ob mice. We also found that differentiation of 3T3-L1 preadipocytes was enhanced by the depletion of PLD1 levels or inhibition of PLD1 activity by VU0155069, a PLD1-specific inhibitor. Conversely, treatment with phosphatidic acid (PA), a PLD product, and overexpression of PLD1 both caused a decrease in adipogenic differentiation. Moreover, the elevated differentiation in PLD1-knockdown 3T3-L1 cells was reduced by either PA treatment or PLD1 expression, confirming negative roles of PLD1 and PA in adipogenic differentiation. Further investigation revealed that PA displaces DEP domain-containing mTOR-interacting protein (DEPTOR) from mTORC1, which subsequently phosphorylates insulin receptor substrate-1 (IRS-1) at serine 636/639 in 3T3-L1 cells. Taken together, our findings provide convincing evidence for a direct role of PLD1 in adipogenic differentiation by regulating IRS-1 phosphorylation at serine 636/639 through DEPTOR displacement and mTOR activation.

Obesity is defined by excessive accumulation of white adipose tissue above the normal level of adipocyte differentiation owing to an energy imbalance. Dietary changes towards high protein and high fat intake have raised the prevalence of obesity over the last decade, increasing the risk of many disorders such as diabetes mellitus, hyperlipidemia, insulin resistance, cardiovascular disease, and cancer^1^,^2^,^3^. An understanding of the molecular mechanisms that regulate adipogenesis is required to reduce obesity and the accompanying susceptibility to many diseases. Adipocyte differentiation is a well-controlled process regulated by an elaborate network of transcription factors, including the CCAAT/enhancer-binding proteins C/EBPβ, C/EBPδ, C/EBPα and peroxisome proliferator-activated receptor γ (PPARγ)^4^,^5^. Expression of C/EBPβ and C/EBPδ is induced in preadipocytes during very early differentiation. Subsequently, these regulators activate PPARγ and C/EBPα, which upregulate each other and maintain their expression to govern the entire adipogenic process by activating additional transcription factors^4^.

The mammalian target of rapamycin (mTOR) pathway regulates many cellular and developmental processes by responding to growth factors and nutrients^6^. mTOR forms two distinct complexes: mTOR complex1 (mTORC1) and mTOR complex2 (mTORC2)^7^. mTORC1 controls translation and protein synthesis by phosphorylating ribosomal S6 kinase 1 (S6K1) and eukaryotic initiation factor 4E binding protein 1 (4EBP1), whereas mTORC2 activates Akt, serum/glucocorticoid-regulated kinase (SGK), and protein kinase Cα (PKCα)^8^,^9^. Recently, a number of studies have shown that mTOR is involved in adipogenesis and lipid metabolism^10^,^11^,^12^,^13^. Inhibition of mTORC1 by either rapamycin treatment or an adipose-specific knockout of regulatory-associated protein of mTOR (also known as RPTOR or raptor, a major component of mTORC1), inhibits adipogenesis^11^,^14^. Conversely, activation of mTORC1 enhances adipogenesis by increasing PPARγ^13^, confirming a positive role for mTOR in adipogenesis^15^. Despite this, mTOR also maintains homeostasis of adipogenesis by suppressing the expression of PPARγ through insulin receptor substrate-1 (IRS-1)/Akt signaling^16^,^17^, suggesting an indispensable function for mTOR in adipogenesis.

Phospholipase D (PLD) hydrolyzes phosphatidylcholine (PC) to yield phosphatidic acid (PA) and choline^18^. PA is a critical regulator of mTOR signaling^19^. Of the several enzymes that are involved in PA biogenesis, PLD1 is the enzyme responsible for activating mTOR via either mitogen or amino acid stimulation^19^,^20^,^21^,^22^. PLD-produced PA is unique in that it contains fatty acid chains with one or two degrees of unsaturation^23^. Although generally PA is reported to maintain mTOR complexes in a steady-state condition^24^, PLD1-produced PA has been shown to specifically bind to the FRB domain of mTOR^19^ and displace an endogenous mTOR inhibitor, DEP domain-containing mTOR-interacting protein (DEPTOR), to activate mTORC1^25^. In addition to the known role of PA in mTOR signaling, it has been suggested that PA regulates epidermal growth factor receptor (EGFR) trafficking from the membranes towards the nucleus and vice versa, and regulates EGFR expression in the nucleus, thus controlling EGF signaling^26^. PA is also involved in neuronal and endocrinal secretion by synaptic vesicle release. The biophysical properties of the PA molecule generate a negative curvature in the inner membrane leaflet of the plasma membrane and subsequently facilitate exocytosis^27^.

PLD has been implicated in a variety of cellular processes, such as the reorganization of actin cytoskeleton, membrane trafficking, secretion, receptor signaling, and differentiation^18^,^28^,^29^,^30^. Several studies using PLD-deficient mice have revealed physiological roles of PLD. Brain development, cognitive funtion^31^, and protection from thrombosis and ischemic stroke^32^ are all impaired in PLD-deficient mice. Mice lacking PLD1 have defects that result in tumor growth and metastasis^33^, and defects in macroautophagy^34^. Even though recent study has shown that PLD deficiency promotes adiposity by up-regulating appetite^35^, the role of PLD/PA in adipogenic differentiation remains unclear. Furthermore, it is not known whether PLD/PA is involved in the autonomous role of mTOR in adipogenesis, despite the fact that PLD and PA are well established as critical regulators of mTOR.

Here, we report that PLD1 plays a negative role in adipogenic differentiation. We observed that PLD1 and PA inhibit the initiation of adipogenesis through activation of mTORC1 via displacement of DEPTOR from mTORC1. We found that PA-activated mTORC1 preferentially phosphorylates IRS-1 at serine 636/639 and regulates the IRS-1/Akt signaling pathway. These results present PLD1 as a newly identified regulator of adipogenesis.

## Results

### PLD1 activity decreases during adipogenesis

**Figure 1:**
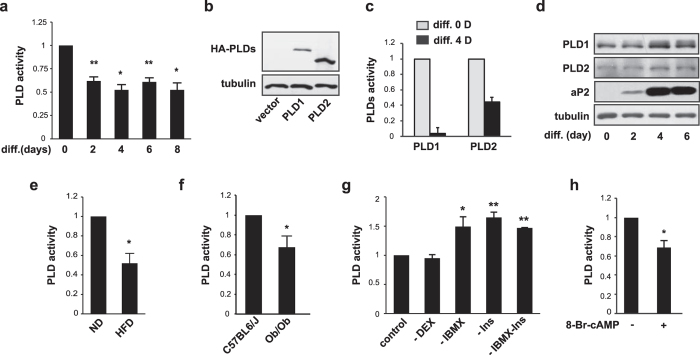
PLD activity is decreased during 3T3-L1 adipogenic differentiation. (**a**) 3T3-L1 cells were induced to differentiate for eight days. On day 0, 2, 4, 6 or 8 of differentiation, cells were lysed and a PLD assay was performed using a PLD assay kit. (**b**,**c**) (**b**) Recombinant PLD1 or PLD2 was transiently expressed. (**c**) Cells were differentiated for four days and recombinant *in vivo* PLD activities were measured using a transphosphatidylation assay as described in Materials and Methods. (**d**) Cells were treated as described in (**a**) and the lysates were subjected to western blotting. (**e**) White adipose tissue (WAT) was isolated from C57BL/6 J mice fed either a normal diet (ND) or a high-fat diet (HFD) for 12 weeks, lysed and subjected to PLD assay using a PLD assay kit (n = 4 per group). (**f**) WAT was isolated from either C57BL/6 J mice or ob/ob mice at age of 12 weeks and then subjected to PLD assay using a PLD assay kit (n = 9–10 per group). (**g**) Cells were differentiated in media under various conditions for four days and then *in vivo* PLD activities were measured with a transphosphatidylation assay. (**h**) 3T3-L1 cells were treated with 100 μM of 8-Br-cAMP and *in vivo* PLD activity was measured as in (**g**). (**a**–**h**) Data are representative of three to four independent experiments. Data are mean ± SD, with paired *t*-tests performed as indicated, *P < 0.05, **P < 0.01.

We set out to test the activity and expression of PLD1 during adipogenic differentiation to examine its role in this process. 3T3-L1 preadipocytes were stimulated to differentiate by addition of differentiation medium 1 (DM1) with dexamethasone, IBMX, and insulin for two days, and then with differentiation medium 2 (DM2) for the following 4–6 days. The accumulation of lipid droplets was noticeable 3–4 days after induction, and adipocytes were fully differentiated after 7–8 days (Supplementary Fig. 1a). The mRNA levels of adipogenic markers (PPARγ, C/EBPα, adipocyte protein 2 (aP2)), and lipogenic markers (fatty acid synthase (FAS) and lipoprotein lipase (LPL)) were increased during differentiation (Supplementary Fig. 1b), as previously reported^4^,^36^,^37^. Measurements showed that total PLD activity during differentiation began to decrease on day 2 (Fig. 1a), and by day 4 PLD activity had dropped to almost 50% of the level seen in undifferentiated adipocytes (measured by 2 independent techniques; Fig. 1a and Supplementary Fig. 2a). Moreover, activity of the PLD isoform, PLD1, almost completely decreased by day 4, whereas the PLD2 activity was still present (Fig. 1b,c). This suggests that PLD1 is responsible for the majority of the decreased PLD activity observed during adipogenic differentiation. We observed that the expression of PLD1 and PLD2 increased slightly during adipocyte differentiation (Fig. 1d), implying that the decrease in PLD activity was not due to a decrease in PLD expression. Importantly, PLD activity decreased in white adipose tissue (WAT) of both high fat diet (HFD)-fed mice (Fig. 1e) and ob/ob mice (Fig. 1f), without any decrease in PLD1 mRNA expression (Supplementary Fig. 2b,c). These results imply that a reduction in PLD activity might be required for adipogenic differentiation.

We proceeded to investigate if a component of the differentiation media was responsible for the decrease in PLD activity. We removed each component from the differentiation media individually, attempted to induce adipocyte differentiation with the resulting media iterations, and then measured PLD activity in each media environment. Removal of either IBMX or insulin increased PLD activity, whereas removal of dexamethasone had no effect on PLD activity (Fig. 1g). Although removal of any one component in the differentiation media decreased the overall rate of differentiation, the decreases did not correlate entirely with the changes observed in PLD activity (Supplementary Fig. 2d). Most notably, while removing insulin and IBMX together resulted in a level of PLD activity similar to that achieved by removing insulin or IBMX alone (last lane in Fig. 1g), the same simultaneous depletion caused a synergistic decrease in differentiation (last lane of Supplementary Fig. 2d). These results suggest that IBMX and insulin are responsible for the decrease in PLD activity, probably via the same signaling pathway, during 3T3-L1 differentiation. IBMX is a phosphodiesterase inhibitor that increases cAMP levels to strongly promote the initiation of adipogenic differentiation^38^. For this reason, we speculated that cAMP decreases PLD activity during adipogenesis; this was further supported by our observation that treating 3T3-L1 preadipocytes with cAMP decreased PLD activity (Fig. 1h), implying that PLD activity is affected by cAMP levels during adipogenesis.

### PLD1 negatively regulates adipogenic differentiation

**Figure 2:**
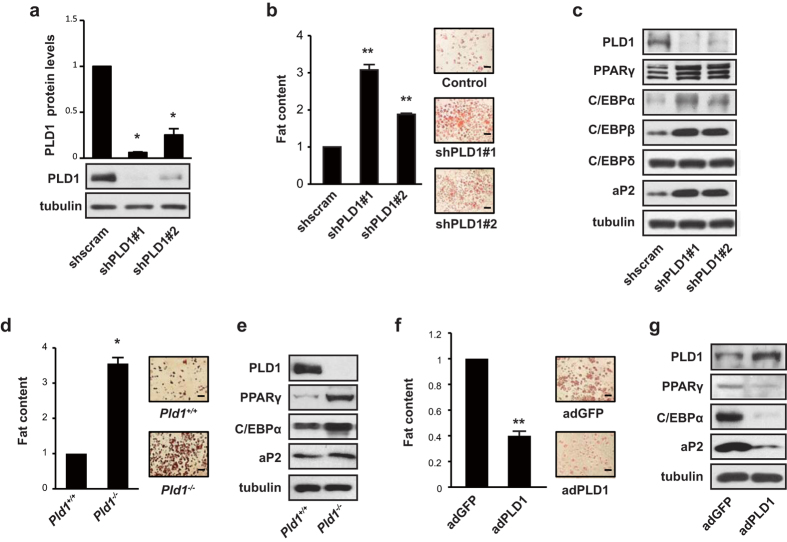
PLD1 negatively regulates adipogenic differentiation. (**a**) 3T3-L1 cells were infected with lentiviruses expressing two different PLD1 shRNAs or scrambled shRNA, and then selected by puromycin for 5 days. Cells were lysed and subjected to western blotting. Western blot intensities were analyzed using ImageJ. (**b**,**c**) Cells were treated as described in (**a**) and induced into differentiation for eight days. Cells were either (**b**) stained with Oil Red O and quantified for lipid contents, or (**c**) lysed and subjected to western blotting. (**d**,**e**) *Pld1*^+/+^ and *Pld1*^−/−^ MEFs were differentiated for eight days and either (**d**) subjected to staining with Oil Red O and quantified for lipid contents, or (**e**) lysed and analyzed by western blotting. (**f**,**g**) Cells were induced to differentiate three days after the infection of adGFP or adPLD1, followed by either (**f**) Oil Red O staining and quantification for lipid content, or (**g**) lysed and analyzed by western blotting. (**a**–**g**) Images are representative of three to five experiments. Scale bars, 100 μm. All data shown are mean ± SD or blots representative of three to five independent experiments. Student *t*-tests were performed to compare the indicated pairs of data. *P < 0.05; **P < 0.01.

To further assess the role of PLD1 in adipogenesis, we performed a PLD1 knockdown using lentiviral shRNA delivery in 3T3-L1 cells. Two PLD1-targeting shRNA species decreased PLD1 protein levels by 94% and 75%, respectively (Fig. 2a). When these two PLD1-targeting shRNAs were expressed in 3T3-L1 cells, lipid droplet formation was significantly enhanced, as shown by Oil Red O staining (Fig. 2b). Quantification of Oil Red O staining revealed an increase of 3.07-fold and 1.88-fold in two PLD1 knockdown samples (Fig. 2b). Correspondingly, PLD1 deficiency resulted in an increase in mRNA levels of PPARγ, C/EBPα, aP2, and lipogenic genes such as LPL and FAS (Supplementary Fig. 3a), followed by elevated expression levels of PPARγ, C/EBPα, C/EBPβ, and aP2 protein upon differentiation (Fig. 2c). We then conducted similar experiments on mouse embryonic fibroblasts (MEFs) that were isolated from 13–15 day embryos of *Pld1*^−/−^ mice. MEFs were induced to differentiate by adding rosiglitazone in DM1. *Pld1*^−/−^ MEFs had more lipid droplets compared to *Pld1*^+/+^ MEFs, as shown by Oil Red O staining and its quantification (Fig. 2d). *Pld1*^−/−^ MEFs also displayed clear increases in levels of the adipogenic differentiation markers PPARγ, C/EBPα, and aP2 over those seen in *Pld1*^+/+^ MEFs (Fig. 2e). Conversely, when we overexpressed PLD1 using adenoviral gene transfer in 3T3-L1 cells, lipid droplet formation on day 8 of differentiation was attenuated by up to 60% compared to control cells (Fig. 2f). Subsequently, mRNA levels of PPARγ, C/EBPα, aP2, and lipogenic genes such as FAS and LPL were decreased (Supplementary Fig. 3b), leading to reduced expression of PPARγ, C/EBPα, and aP2 protein (Fig. 2g). These results suggest that PLD1 is a critical negative regulator of adipogenesis.

### Inhibition of PLD1 activity induces adipogenesis

**Figure 3:**
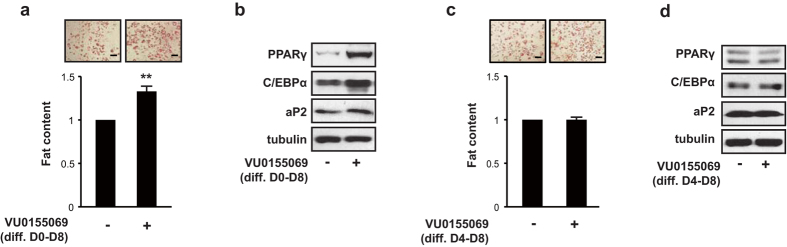
Inhibition of PLD1 activity induces adipogenic differentiation. (**a**,**b**) 3T3-L1 cells were differentiated in the presence of 5 μM VU0155069 at the initiation of differentiation for eight days. Cells were either (**a**) fixed and stained with Oil Red O and quantified for lipid content, or (**b**) lysed and analyzed by western blotting. (**c**,**d**) 3T3-L1 cells were induced to differentiate and 5 μM VU0155069 was added on day 4 of differentiation for four days. Cells were either (**c**) stained with Oil Red O and quantified for lipid content, or (**d**) lysed and analyzed by western blotting. (**a**–**d**) Images are representative of three to five experiments. Scale bars, 100 μm. All data shown are mean ± SD or blots representative of three to five independent experiments. Student *t*-tests were performed to compare the indicated pairs of data. **P < 0.01.

To address whether the enzymatic activity of PLD1 is required for the inhibitory effect of PLD1 during adipogenesis, we utilized VU0155069, a PLD1-specific inhibitor. Treatment of cells with VU0155069 upon induction of adipogenic differentiation increased lipid droplet formation by up to 36% compared to vehicle-treated cells (Fig. 3a). This is consistent with our observations in PLD1-deficient 3T3-L1 cells and *Pld1*^−/−^ MEFs, specifically that PLD1 deficiency increased lipid accumulation. In addition, expression of adipogenic differentiation markers PPARγ, C/EBPα, and aP2 were elevated in VU0155069-treated cells (Fig. 3b and Supplementary Fig. 4), indicating that PLD1 negatively regulates adipogenesis. When VU0155069 was added for four days after day 4 of differentiation, adipogenic differentiation was not enhanced, as shown by lipid droplet formation (Fig. 3c) and expression of PPARγ, C/EBPα, and aP2 protein (Fig. 3d). These results suggest that PLD1 acts at the beginning of adipogenic differentiation, rather than during terminal differentiation or during maintenance of adipogenic characteristics in mature adipocytes.

### PA, a PLD1 product, inhibits adipogenesis

**Figure 4:**
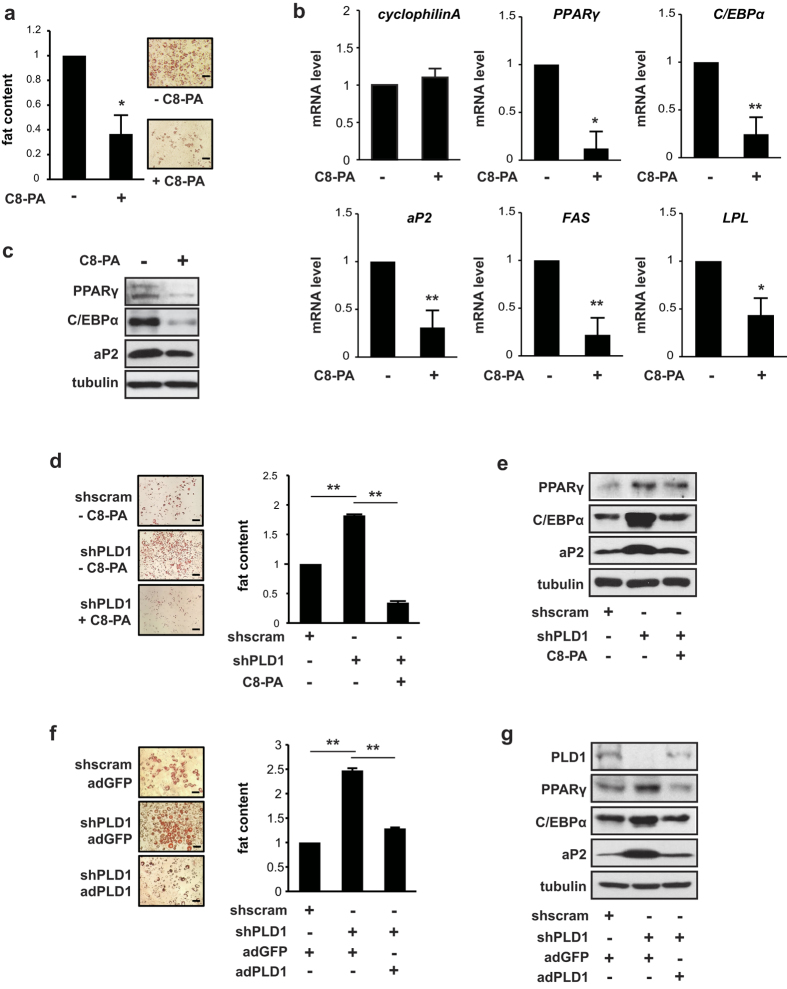
PA, the product of PLD1, inhibits adipogenesis. (**a**) 3T3-L1 preadipocytes were differentiated with 300 μM C8-PA at the initiation of differentiation for eight days, followed by staining with Oil Red O and quantified for lipid content. (**b**,**c**) Cells were treated as described in (**a**), and then analyzed by either (**b**) quantitative RT-PCR or (**c**) western blotting. Mouse GAPDH was used to normalize gene expression. Cyclophilin A was used as an internal control. (**d**) 3T3-L1 cells were infected with either shscramble or shPLD1 and selected with puromycin for three days. Cells were induced to differentiate with or without 300 μM C8-PA. After eight days of differentiation, cells were fixed and stained with Oil Red O and quantified for lipid content. (**e**) Cells were treated as described in (**d**) and analyzed by western blotting. (**f**) Cells were infected with adenoviral PLD1 for three days in shPLD1-infected cells. Cells were then differentiated for eight days, followed by Oil Red O staining and quantification of lipid content. (**g**) Cells were treated as described in (**f**) and analyzed by western blotting. (**a**–**g**) Images are representative of three to five experiments. Scale bars, 100 μm. All data shown are mean ± SD or are blots representative of three to five independent experiments. Student *t*-tests were performed to compare the indicated pairs of data. *P < 0.05; **P < 0.01.

In order to determine whether phosphatidic acid (PA), a PLD1 product, is required for inhibition of adipogenic differentiation, we treated cells with a short chain PA (C8-PA) when differentiation was induced. We chose to use this PA species because it is not converted into active lysophosphatidic acid that could otherwise initiate signaling through membrane bound lysophosphatidic acid receptors^39^. As shown in Fig. 4a, PA treatment drastically decreased lipid formation, as shown by Oil Red O staining, and quantification revealed a reduction of up to 63% compared to control cells. Expression of PPARγ, C/EBPα, aP2 and lipogenic genes such as FAS and LPL was also reduced (Fig. 4b,c), which is consistent with the inhibitory effect of PLD1 expression on adipogenic differentiation. Interestingly, the augmented adipogenic differentiation in shPLD1-infected 3T3-L1 cells was reduced by either treatment with PA or by PLD1 expression via adPLD1 (adenovirus vector carrying the gene for PLD1), as shown by lipid content (Fig. 4d,f), mRNA levels of adipogenic and lipogenic markers such as PPARγ, C/EBPα, aP2, LPL and FAS (Supplementary Fig. 5a,b), and protein levels of PPARγ, C/EBPα, and aP2 (Fig. 4e,g). These results imply that PLD1 suppresses adipocyte differentiation through its enzymatic product, PA.

### PA regulates adipogenic differentiation by displacing DEPTOR from mTORC1

**Figure 5:**
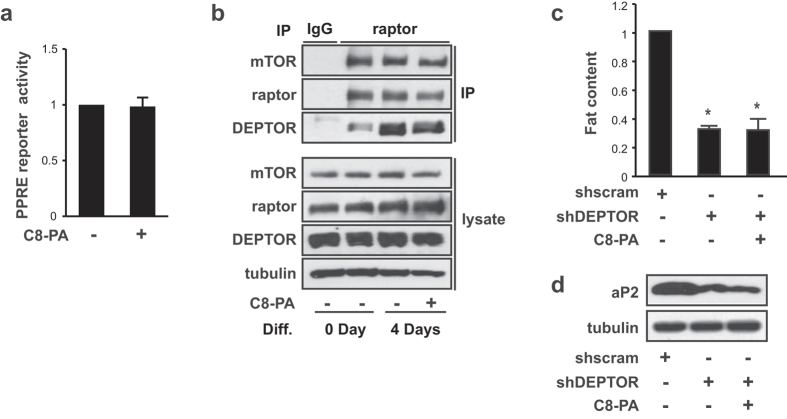
PA regulates adipogenic differentiation by displacing DEPTOR from mTORC1. (**a**) 3T3-L1 preadipocytes were transfected with a PPRE luciferase reporter plasmid vector, induced to differentiate for 3 h, and then luciferase activity was measured. (**b**) 3T3-L1 cells were differentiated in the absence or presence of 300 μM C8-PA. Cell lysates and immunoprecipitates against raptor were analyzed by western blotting. (**c**,**d**) 3T3-L1 cells were infected by either shscramble or shDEPTOR and selected with puromycin for five days. After eight days of differentiation with or without 300 μM C8-PA, cells were subjected to either (**c**) Oil Red O staining and quantification of lipid content, or (**d**) western blotting. (**a**–**d**) All data shown are mean ± SD or are blots representative of three to five independent experiments. Student *t*-tests were performed to compare the indicated pairs of data. *P < 0.05.

Since PA decreased the expression of adipogenic genes during adipogenic differentiation, we considered it possible that PA directly regulates PPARγ activity—which itself coordinates expression of many genes responsible for the establishment of mature adipocytes^40^. To test this idea, we transfected 3T3-L1 adipocytes with a luciferase reporter that contained triple repeats of the DR1 sequence, a potent PPRE (PPARγ responsive element) and then induced differentiation. As shown in Fig. 5a, PA did not affect PPARγ activity. This suggests that PA regulates differentiation not by directly influencing PPARγ activity, but rather through a PA-induced signaling mechanism.

PLD1-produced PA, which contains at least one unsaturated fatty acid, displaces DEPTOR, an endogenous mTORC1 inhibitor, from mTORC1 following mitogenic stimulation, resulting in mTORC1 activation^25^. DEPTOR expression is increased in WAT in an obese mouse model^17^. Moreover, DEPTOR enhances adipogenesis through Akt activation via mTORC1-induced phosphorylation of IRS-1 at serine 636/639, which is followed by PPARγ activation^17^. Therefore, in order to identify the mechanisms that underlie the inhibitory function of PA, we examined whether PA was able to displace DEPTOR from mTORC1 in 3T3-L1 cells. As shown in Supplementary Fig. 6, the level of DEPTOR in mTORC1 was significantly reduced in 3T3-L1 preadipocytes after 30 min treatment with PA. This is consistent with a previous report^25^. Notably, the level of DEPTOR in mTORC1 was increased during differentiation in 3T3-L1 cells (Fig. 5b; the second lane versus the third lane). PA treatment during differentiation reduced the level of DEPTOR in mTORC1 (Fig. 5b; the third lane versus last lane). In addition, DEPTOR knockdown by lentiviral delivery in 3T3-L1 cells drastically diminished lipid droplet formation as shown by Oil Red O staining and quantification (Supplementary Fig. 7a), and it reduced the expression of adipogenic differentiation markers such as PPARγ, C/EBPα, and aP2 (Supplementary Fig. 7b), confirming that DEPTOR is a positive regulator of adipogenesis (this is in agreement with a previous report)^17^. Since PA displaced DEPTOR from mTORC1 (Fig. 5b), we speculated that PA would have no further inhibitory effect on adipogenic differentiation in DEPTOR-knockdown 3T3-L1 cells, and we were able to confirm this experimentally. PA treatment did not further decrease adipogenic differentiation in DEPTOR-depleted cells, as shown by quantification of lipid content (Fig. 5c) and aP2 expression, a terminal differentiation marker (Fig. 5d). These results indicate that PA inhibits adipogenic differentiation via displacement of DEPTOR from mTORC1.

### PA inhibits adipogenesis through the IRS-1/Akt pathway

**Figure 6:**
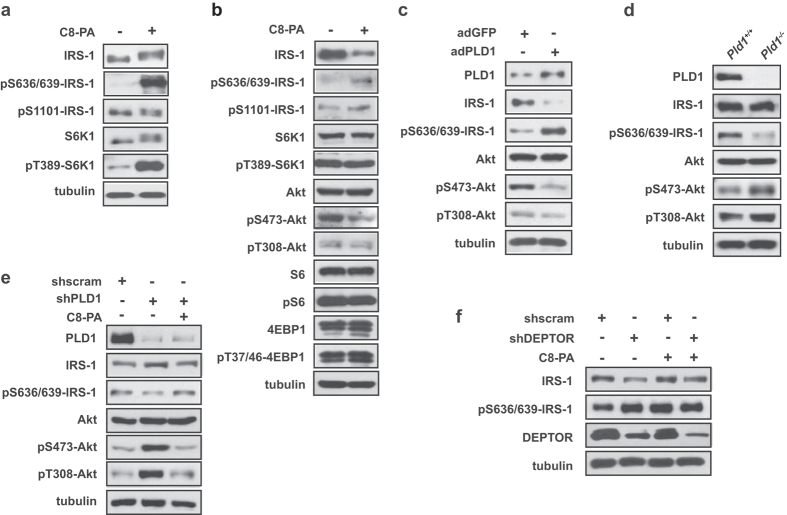
PA inhibits adipogenesis through the IRS-1-Akt axis. (**a**) 3T3-L1 preadipocytes were serum-starved overnight, then stimulated with 300 μM C8-PA for 30 min. Cell lysates were analyzed by western blotting. (**b**) 3T3-L1 cells were induced to differentiate either with or without 300 μM C8-PA for eight days, then lysed and subjected to western blotting. (**c**) 3T3-L1 cells were infected with either adGFP or adPLD1, induced to differentiate for eight days, and then analyzed by western blotting. (**d**) *Pld1*^+/+^ and *Pld1*^−/−^ MEFs were differentiated for eight days, lysed and analyzed by western blotting. (**e**) Cells were infected with either shscramble or shPLD1 #1, selected with puromycin for five days, then induced to differentiate either with or without 300 μM PA for eight days, followed by analysis by western blotting. (**f**) 3T3-L1 cells were infected with either shscramble or shDEPTOR and treated either with or without 300 μM PA for 30 min, then lysed and analyzed by western blotting. (**a**–**f**) All data shown are blots representative of three to five independent experiments.

To better understand the suppressive role of PA in adipogenesis, we determined the effect of PA on mTORC1 signaling in 3T3-L1 cells. PA has been reported to selectively regulate mTORC1 activity *in vivo* and *in vitro*^25^,^39^. As shown in Fig. 6a, treatment with PA for 30 minutes robustly increased IRS-1 phosphorylation at serine 636/639, without affecting phosphorylation at serine 1101. IRS1 phosphorylation at serine 307 was not detected in 3T3-L1 cells (data not shown). IRS1 phosphorylation at serine 636/639 is directly targeted by mTORC1 and induces IRS-1 degradation^41^. In addition, either PA treatment for eight days, or PLD1 overexpression by adenoviral PLD1 during 3T3-L1 adipogenic differentiation, increased S636/639-IRS-1 phosphorylation, which was followed by decreases in IRS-1 protein levels and by a decrease in Akt phosphorylation at serine 473 and threonine 308 (Fig. 6b,c). Notably, PA treatment did not affect any other classical mTORC1 downstream targets (S6K1, S6, 4EBP1) (Fig. 6b), indicating that, during adipogenesis, PLD1/PA does not regulate mTORC1 in the same manner as in other biological contexts. Conversely, depletion of PLD1 reduced S636/639-IRS-1 phosphorylation and increased Akt phosphorylation at S473 and T308 on day 8 of differentiation in either *Pld1*^−/−^ MEFs (Fig. 6d) or shPLD1-infected cells (Fig. 6e). PA treatment restored S636/639-IRS-1 phosphorylation and decreased Akt phosphorylation in PLD1 knockdown 3T3-L1 cells (Fig. 6e; the second lane versus the third lane). However, PA treatment did not cause a further increase in S636/639-IRS-1 phosphorylation in DEPTOR-depleted cells, in which S636/639-IRS-1 phosphorylation was already increased (Fig. 6f). These results indicate that PA induces IRS-1 phosphorylation via displacement of DEPTOR from mTORC1.

## Discussion

Adipogenic differentiation is a cellular and developmental process that is important for metabolic homeostasis. In the present study, we provide evidence that PLD1 regulates adipogenic fat accumulation. In the early stages of adipogenesis, a decrease in PLD1 activity elevates adipogenic differentiation by up-regulating DEPTOR levels in mTORC1, and consequently, down-regulating mTORC1 activity. In describing this regulatory pathway, we identified PLD1 as a negative regulator of adipogenesis.

PLD activity was previously reported to be indispensable for lipid droplet formation by enhancing ERK2-induced dynein phosphorylation^42^,^43^, pointing to a role for PA in lipid metabolism. In order to maintain phospholipid flux, PA is converted to diacylglycerol (DAG), which is further metabolized into either triglycerides (TG) or phosphatidylcholine (PC)^44^, followed by enhanced lipid accumulation. However, in the present study, either PLD overexpression or PA treatment resulted in decreased rates of adipogenic differentiation, suggesting that PLD1 does not metabolize lipids and form lipid droplets during adipogenic differentiation. We found that PLD activity was reduced by both IBMX and insulin (Fig. 1g). IBMX inhibits phosphodiesterases, resulting in an increase in cellular cAMP^5^. Upon adipogenic differentiation, high cAMP levels lead to activation of C/EBPα and PPARγ, and also drive aP2 expression^45^,^46^. Previous reports showed that cAMP or cAMP-increasing agents inhibit PLD activity *in vitro* and *in vivo*^47^,^48^,^49^. In agreement with these reports, we observed that cAMP decreased PLD activity (Fig. 1h), suggesting that PLD is a downstream target of cAMP in adipogenesis. Unlike cAMP, insulin was reported to activate PLD activity in 3T3-L1 cells, which peaked within the first 2 minutes and then returned to basal levels^50^. It is therefore plausible that the decreased PLD activity we observed in the present study four days after differentiation may not be a direct effect of insulin on PLD activity. Supporting this idea, cotreatment with IBMX and insulin decreased PLD activity to an extent similar to treatment with either IBMX alone or insulin alone, suggesting that IBMX and insulin regulate PLD activity through the same pathway, possibly regulating cAMP levels. The mechanism by which insulin regulates PLD activity during 3T3-L1 differentiation requires further investigation.

PLD activity was decreased in both HFD-induced obese mice (Fig. 1e) and ob/ob mice (Fig. 1f), indicating that reduction of PLD activity correlates with obesity in physiological contexts. Recently, the role of PLD1 and PLD2 has been implicated in protecting against excessive weight gain^35^. Mice lacking either PLD1 or PLD2 consumed more food compared to control mice; this was accompanied by the expression of neuropeptides controlling food intake in the hypothalamus. Subsequently, mice lacking either PLD1 or PLD2 exhibited elevated body weight and adipose tissue content. Although this finding supports that PLD1 is a negative regulator of adipogenesis, it is most unlikely that PLD deficiency elicits obesity simply due to a defect in adipose tissue. Studies of adipose-tissue-specific PLD1 knockout mice are needed to confirm our observations *in vivo*, and to assess any adipose tissue-specific contribution to obesity.

PA is known to prompt signaling events, such as mTOR signaling^19^,^25^. The role of mTOR signaling in adipogenesis is complex, involving a balance of positive and negative regulatory functions. Through the present study, we clarified one piece of this puzzle by demonstrating that the upstream regulators, PLD1 and PA, preferentially regulate mTORC1’s negative function in adipogenic differentiation. During 3T3-L1 differentiation, PLD1/PA activates mTORC1 by displacing DEPTOR and phosphorylating S636/639-IRS-1, which is a direct target of mTORC1 and promotes IRS-1 degradation and inhibition of adipogenesis. Notably, PA did not augment the phosphorylation of S6K1/S6 and 4EBP1—two well-known downstream effectors of mTORC1—during adipogenesis. These results suggest that mTORC1 activation by DEPTOR displacement in differentiating adipocytes selectively induces the negative feedback on IRS-1, without affecting mTORC1 regulation of other substrates. In contexts where mTORC1 and, therefore, S6K1 are completely inhibited, there is a subsequent decrease of S6K1-facilitated adipogenic gene expression. This precludes any significant positive contribution of the IRS-1/Akt pathway to adipogenesis, despite the fact that that pathway is no longer inhibited by mTORC1^16^,^17^. Conversely, partial inhibition of mTOR, which relieves the direct phosphorylation and negative regulation of IRS-1 and subsequently increases Akt activity while preserving S6K1 signaling, increases adipogenesis by way of enhanced lipid accumulation and adipogenic marker expression^16^. In the present study, we have identified a third context wherein, in differentiating adipocytes with high levels of DEPTOR, PA preferentially stimulates phosphorylation of S636/639-IRS-1, while leaving T389-S6K1 phosphorylation levels unchanged. It appears that these high DEPTOR expression levels limit the extent of mTORC1 activation by PA, resulting in mild mTORC1 activation that is specific to, and directly responsible for, downregulation of the IRS-1/Akt pathway.

PA has been reported to regulate the membrane association of lipin1β via binding to the polybasic motif of lipin1β, a protein which has dual functions as a factor in intracellular lipid metabolism and in transcriptional control of adipogenesis^44^. Maintenance of the distribution of lipin1β at both the intracellular membrane and within the nucleus is critically important to the biological activity of lipin1β^44^. Given the role of PA in maintaining lipin1β distribution, both PA deficiency and PA increase were expected to inhibit adipogenesis due to a disruption in the balance of lipin1β localization. However, the current study shows that PA deficiency increases adipocyte differentiation while PA treatment decreases it, indicating that the role of PA in adipogenesis is not related to regulation of the balance of lipin1β between the nucleus and cytoplasm.

PLD2, an isoform of PLD, produces cyclic phosphatidic acid (CPA) from lysophosphatidylcholine. CPA directly binds to PPARγ with nanomolar affinity and stabilizes the binding of a corepressor, silencing mediator of retinoid and thyroid hormone receptors (SMRT), to PPARγ — resulting in inhibition of PPARγ-related gene expression^51^,^52^. CPA also inhibits phosphodiesterase 3B (PDE3B) expression and subsequently increases cAMP, leading to increased lipolysis in 3T3-L1 cells and an inhibition of adipogenesis^53^. Even though both PLD1 and PLD2 hydrolyze PC to produce PA, PLD1 is not involved in insulin-stimulated CPA production^52^. Moreover, our results show that PA does not directly modulate PPARγ activity (Fig. 5a), suggesting that PLD1-induced inhibition of adipogenesis is not the result of a direct effect of PA on PPARγ activity.

Notably, expression of PLD1 and PLD2 mildly increased during adipogenic differentiation, despite the fact that PLD functions as a negative regulator. It was reported that the expression levels of negative regulators increased during adipogenic differentiation^54^ as well as during other forms of cellular differentiation, such as myogenesis^55^,^56^. Since PLD/PA controls adipogenesis through several different pathways (e.g., lipin1β localization, PPARγ activity through PLD2-prduced CPA, mTOR signaling through DEPTOR), precise regulation of PLD is required for the fine control of adipogenic differentiation. The increased expression of PLD might introduce a necessary negative feedback loop that prevents excessive differentiation.

In conclusion, we have shown that PLD1 inhibits adipogenesis via specific induction of a negative role for mTORC1. These findings improve our insights into the regulation of PLD1 in mTOR signaling and increase our understanding of the molecular mechanisms that underlie adipogenic differentiation. Furthermore, these new molecular discoveries may prompt the development of PLD1-specific activation methods as a therapeutic strategy for treating obesity.

## Materials and Methods

Cell lysis, immunoprecipitation, western blotting, Oil Red O staining and lipid content assay, and quantitative RT-PCR were all performed following standard procedures or as previously reported, with detailed information described in the Supplementary information.

### Antibodies and other reagents

Antibodies against the following proteins were obtained from the following sources: alpha-tubulin (ab11304) from Abcam; DEPTOR (NBP149674) from Novus Biologicals; raptor (for IP, A300-553A) from Bethyl laboratories; PLD2 (sc-25513), PPARγ (sc-7196), C/EBPα (sc-61) and aP2 (sc-271529) from Santa Cruz Biotechnology; C/EBPβ (#3087), C/EBPδ (#2318), IRS-1 (#2382), pS636/639-IRS-1 (#2388), pS1101-IRS-1 (#2385), mTOR (#2972), raptor (#2280), Akt (#9272), pS473-Akt (#4051), pT308-Akt (#9275), S6K1 (#9202), pT389-S6K1 (#9234), S6 (#2217), pSer235/236-S6 (#4856), 4EBP1 (#9644), and pT37/46-4EBP1 (#2855) from Cell Signaling Technology. The PLD1 antibody was provided by Dr. Jie Chen from the University of Illinois at Urbana-Champaign^16^. All secondary antibodies were from Jackson ImmunoResearch Laboratories Inc. [9,10-^3^H(N)]-oleic acid was from PerkinElmer. C8-PA (830842C) was from Avanti. All other reagents were from Sigma-Aldrich.

### Cell culture

3T3-L1 preadipocytes were grown at 37 °C in 5% CO_2_ in Dulbecco’s modified Eagle’s medium (DMEM) containing 4.5 g/L glucose and 10% bovine calf serum (M1). For 3T3-L1 preadipocyte differentiation, 2 days after the cells were confluent in M1 (day 0), the cells were induced to differentiate in M2 (DMEM containing 10% fetal bovine serum [FBS], 1.0 μM dexamethasone, 0.5 mM IBMX, and 1.5 μg/mL insulin). After 48 hours, M2 medium was replaced by M3 (DMEM containing 10% FBS and 1.5 μg/mL insulin), and cells were fed every 2 days with M3. At the time points indicated, cells were harvested for RT-PCR, western blotting, or Oil Red O staining. *Pld1*^−/−^ mice were generated and characterized as described^34^. To prepare *Pld1*^+/+^ and *Pld1*^−/−^ MEFs, 13–15 day post-coital mouse embryos were minced and digested with trypsin. The cells were collected and cultured in DMEM with 4.5 mg/L glucose, 10% FBS, 100 U/mL penicillin, and 100 μg/mL streptomycin. MEFs were induced to differentiate by adding rosiglitazone in DM1.

### Animals

For diet-induced obese mice, 7-week-old C57BL/6 J mice were obtained from the Jackson Laboratory. After adaptation for one week, mice were provided with either a HFD (60% fat) or regular chow diet (5.4% fat) for 12 weeks. C57BL/6 J mice and ob/ob mice were obtained from Daehan Bio Link (Daejeon, Korea) and Japan SLC (Hamamatsu, Japan), respectively. Food and water were provided *ad libitum*, and the mice were kept on a 12 h light, 12 h dark cycle. Male mice were used for all studies. All experimental protocols for animals, maintenance and care, were conducted in accordance with Gachon University Animal Care guidelines. All animal procedures were approved by the Gachon University of Medicine and the Institutional Animal Care and Use Committee (IACUC).

### PLD assays

*In vivo* PLD activity was determined by PLD-catalyzed transphosphatidylation as previously described^20^. Briefly, 3T3-L1 cells were metabolically labeled with ^3^H-oleic acid for 18 h. After pretreatment with 0.3% 1-butanol for 30 min, cells were lysed and lipids were extracted according to the method of Bligh and Dyer^57^ and analyzed by TLC. Alternatively, a Phospholipase D Assay Kit (Sigma Aldrich) was also used, as indicated in the figure legends, to measure PLD activity according to the manufacturer’s instructions^58^,^59^,^60^,^61^. To calculate recombinant PLD1 or PLD2 activity specifically, the PLD activity in cells transfected with empty vector was subtracted from the activity in PLD1 or PLD2-transfected cells under the same conditions^20^,^62^. Cells were transfected with HA-PLD1^20^ and HA-PLD2^20^ using Amaxa Nucleofector technology (Amaxa, Cologne, Germany) according to manufacturers’ protocol.

### Lentivirus-mediated shRNA and adenovirus-mediated gene expression

Lentiviral shRNA plasmids were used in the pLKO.1puro vector and lentivirus, and were packaged into 293T cells as previously described^30^. Mouse PLD1 shRNA#1 (TRCN0000076820), mouse PLD1 shRNA#2 (TRCN0000076822), negative control shRNA (scrambled hairpin sequence), mouse DEPTOR shRNA #1 (Addgene 21337), and mouse DEPTOR shRNA #2 (Addgene 21338) were used as previously described^30^,^63^. After viral infection, selection of infected cells with 4 μg/mL puromycin was performed for 3–5 days before differentiation was induced. 3T3-L1 cells were incubated with recombinant adenoviruses bearing human PLD1 cDNA or the GFP gene for 24 h and induced to differentiate eight days after infection. Adenoviral PLD1 and adenoviral GFP were used as previously reported^64^.

### Luciferase assays

Cells were transfected with the PPARγ responsive element (PPRE) luciferase reporter (3x DR1) plasmid vector^14^ using the TransIT-LT1 transfection reagent (Mirus) following manufacturers’ recommendations. Cells were induced into differentiation for 3 h and lysed in Passive Lysis buffer (Promega). Luciferase assays were performed using a Luciferase Assay System kit (Promega) following the manufacturer’s protocol.

### Statistical analysis

All numerical data are presented as mean ± standard deviation (SD). Where necessary, statistical significance was determined by performing one-sample *t*-tests. *P*-values <0.05 were considered statistically significant.

## Additional Information

**How to cite this article**: Song, H.-I. and Yoon, M.-S. PLD1 regulates adipogenic differentiation through mTOR - IRS-1 phosphorylation at serine 636/639. *Sci. Rep*. **6**, 36968; doi: 10.1038/srep36968 (2016).

**Publisher’s note:** Springer Nature remains neutral with regard to jurisdictional claims in published maps and institutional affiliations.

## Supplementary Material

Supplementary Information
