# Supplementary material for: PLD1 regulates adipogenic differentiation through mTOR - IRS-1 phosphorylation at serine 636/639
Source: Sci Rep. 2016 Nov 22;6:36968. doi: 10.1038/srep36968 (PMC5181839; doi:10.1038/srep36968)
Supplement: Supplementary Information [file srep36968-s1.pdf]

SUPPLEMENTARY INFORMATION

PLD1 regulates adipogenic differentiation through mTOR - IRS-1 phosphorylation at serine 636/639

Hae-In Song[1] and Mee-Sup Yoon[1*]

[1]Department of Molecular Medicine, School of Medicine,

Gachon University, Incheon 406-840, Korea.

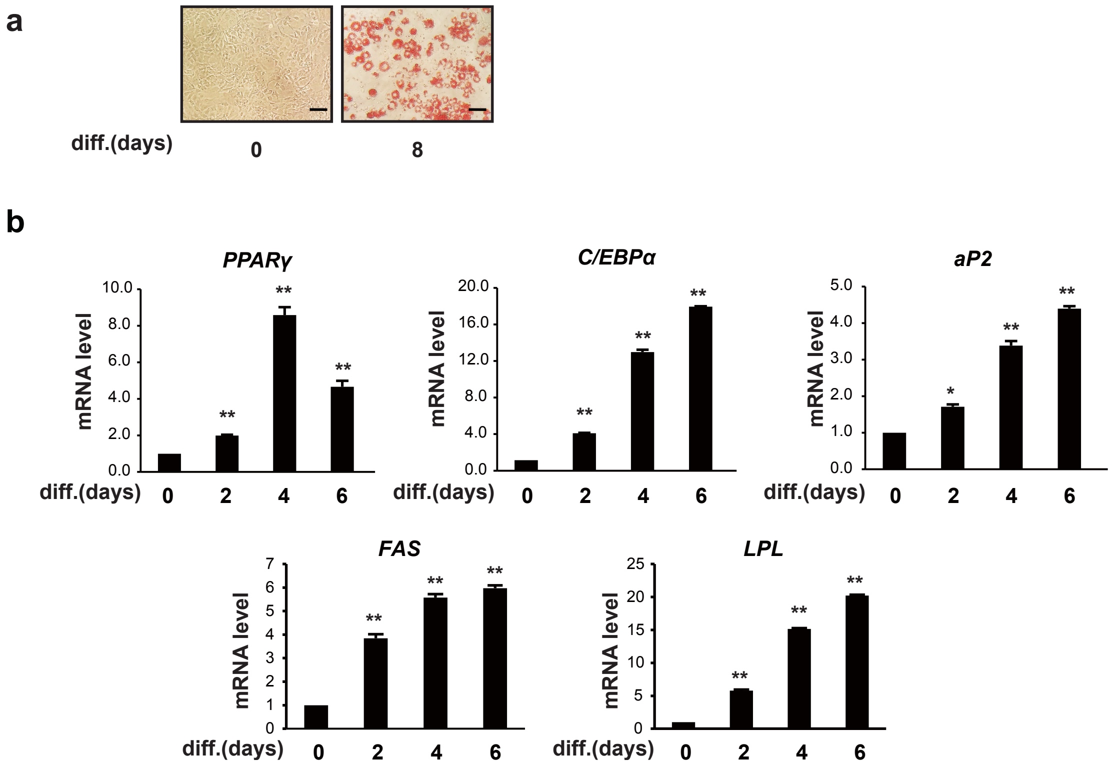

Supplementary Figure 1. Characterization of 3T3-L1 differentiation

(a) 3T3-L1 cells were induced to differentiate for eight days and then stained with Oil Red O. (b) On day 0, 2, 4, or 6 of differentiation, cells were subjected to RNA isolation and quantitative RT-PCR. Mouse GAPDH was used to normalize gene expression. Average results are shown, with error bars representing standard deviation. All data shown are mean ± SD from three to five independent experiments. Student *t*-tests were performed to compare the indicated pairs of data. **P*<0.05; ***P*<0.01. Images are representative of three to five experiments. Scale bars, 100 μm.

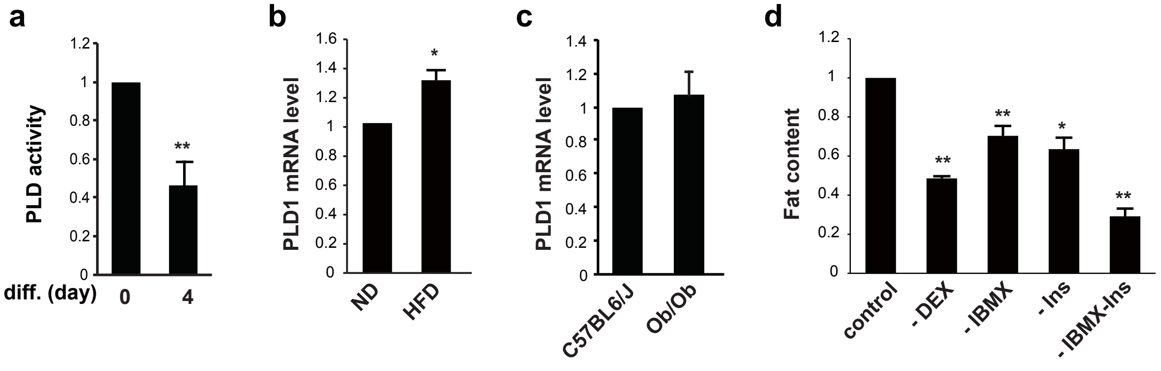

Supplementary Figure 2. PLD1 expression is not decreased in HFD-fed mice and ob/ob mice.

(a) 3T3-L1 cells were induced to differentiate for 4 days and then *in vivo* PLD activities were measured with a transphosphatidylation assay. (b) White adipose tissue (WAT) was isolated from C57BL/6J mice fed either a normal diet (ND) or a high-fat diet (HFD) for 12 weeks, lysed and subjected to quantitative RT-PCR (n=4 per group). (c) WAT was isolated from either C57BL/6J mice or ob/ob mice at age of 12 weeks and then subjected to quantitative RT-PCR (n=9-10 per group). (b-c) Mouse GAPDH was used to normalize gene expression. (d) 3T3-L1 cells were differentiated in media under various conditions for four days, and then fat content was measured. Average results are shown, with error bars representing standard deviation. Data are representative of three to four independent experiments. Data are mean ± SD, with paired *t*-tests performed as indicated, *$P < 0.05$, ** $P < 0.01$.

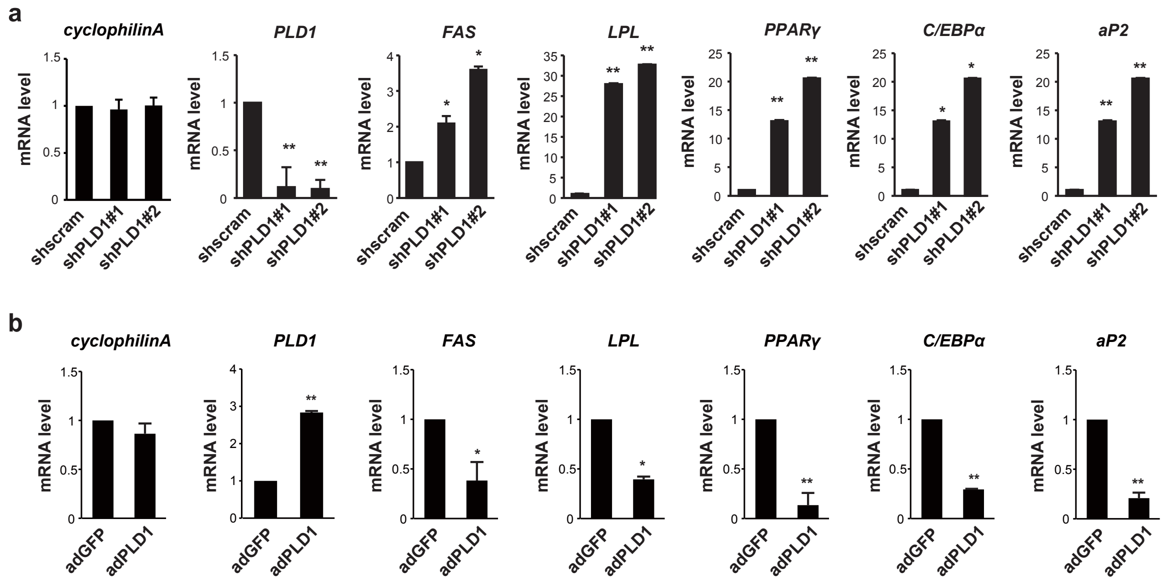

Supplementary Figure 3. PLD1 regulates adipogenesis.

(a) 3T3-L1 cells treated as shown in Fig. 2 (b) were subjected to RNA isolation and quantitative RT-PCR. (b) Cells treated as shown in Fig. 2 (f) were analyzed by quantitative RT-PCR. Mouse GAPDH was used to normalize gene expression. Cyclophilin A was used as an internal control. Average results are shown, with error bars representing standard deviation. All data shown are mean ± SD from three to five independent experiments. Student *t*-tests were performed to compare values within each data set. **P*<0.05; ***P*<0.01.

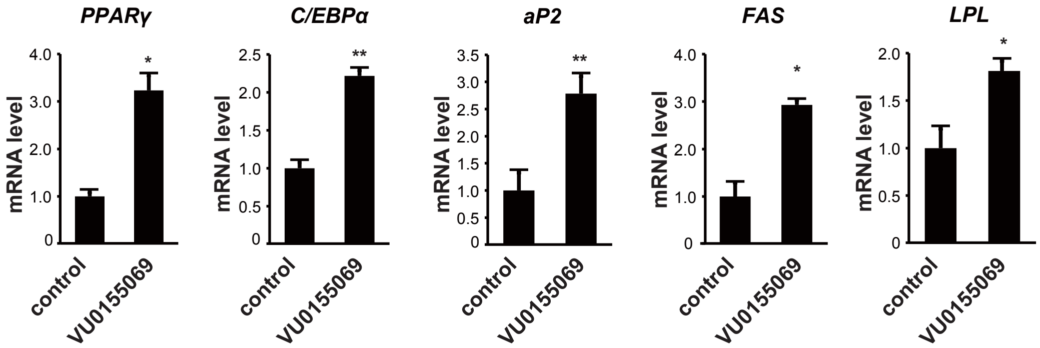

Supplementary Figure 4. Inhibition of PLD1 by VU0155069 increases adipogenesis

Cells treated as shown in Fig. 3 (a) were subjected to RNA isolation and quantitative RT-PCR. Mouse GAPDH was used to normalize gene expression. Average results are shown, with error bars representing standard deviation. All data shown are mean ± SD from three to five independent experiments. Student *t*-tests were performed to compare each data pair. *P<0.05; **P<0.01.

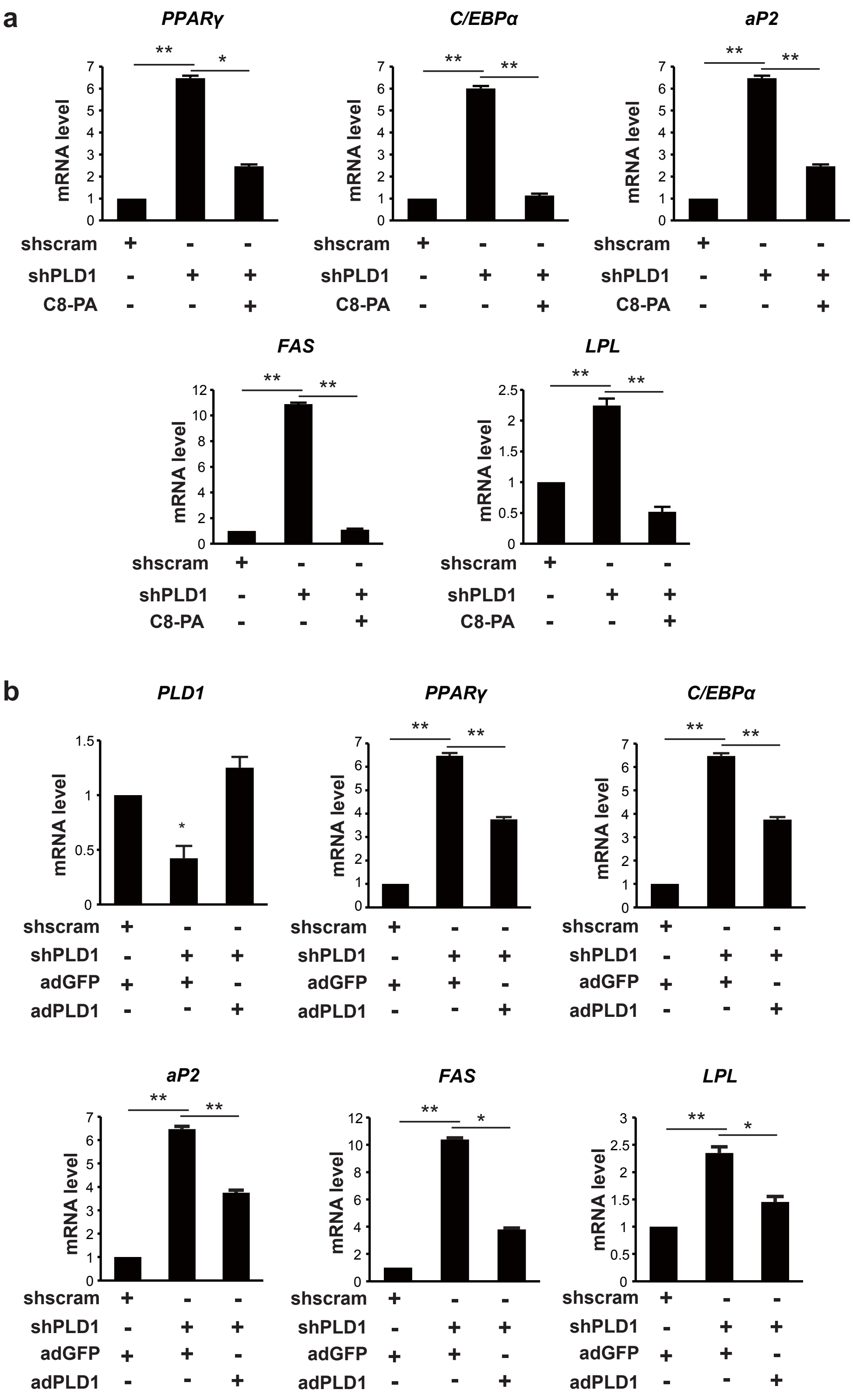
a
PPARγ
C/EBPα
aP2
FAS
LPL
mRNA level
**
*
shscram + - -
shPLD1 - + +
C8-PA - - +
b
PLD1
PPARγ
C/EBPα
aP2
FAS
LPL
mRNA level
shscram + - -
shPLD1 - + +
adGFP + + -
adPLD1 - - +

Supplementary Figure 5. Restoration of PA alleviates enhanced differentiation in PLD1 knockdown cells

(a) 3T3-L1 cells treated as shown in Fig. 4 (d) were subjected to RNA isolation and quantitative RT-PCR. (b) Cells treated as shown in Fig. 4 (f) were analyzed by quantitative RT-PCR. Mouse GAPDH was used to normalize gene expression. Average results are shown, with error bars representing standard deviation. All data shown are mean ± SD from three to five independent experiments. Student *t*-tests were performed to compare the indicated pairs of data. **P*<0.05; ***P*<0.01.

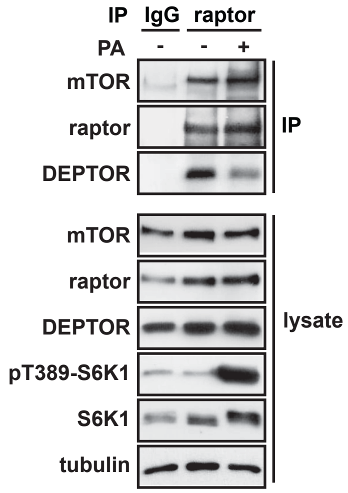

Supplementary Figure 6. PA displaces DEPTOR from mTORC1

3T3-L1 cells were serum-starved overnight, then stimulated with 300 μM C8-PA for 30 min. mTORC1 was isolated by immunoprecipitation of raptor. Cell lysates and immunoprecipitates were analyzed by western blotting. The data shown are from a single experiment that is representative of three to five independent experiments.

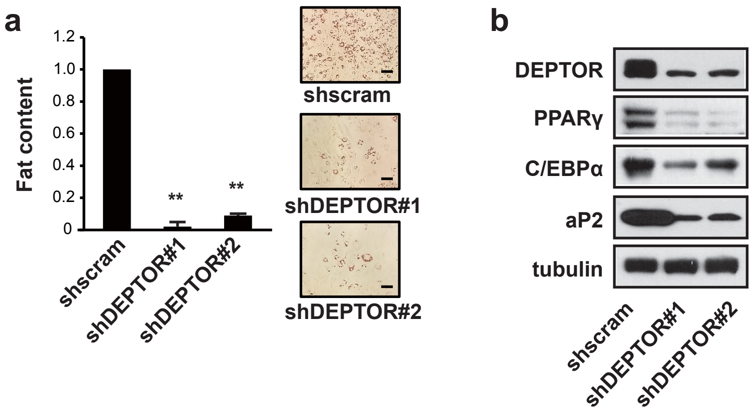

Supplementary Figure 7. DEPTOR is a positive regulator of adipogenesis

(a-b) 3T3-L1 cells were infected by either shscramble or shDEPTOR and selected with puromycin for five days. After eight days of differentiation, cells were subjected to either (a) Oil Red O staining and quantification of lipid content, or (b) western blotting. Images are representative of 3 to 5 experiments. Scale bars, 100 µm. All data shown are mean ± SD or are blots representative of three to five independent experiments. Student *t*-tests were performed to compare the knockdown samples to the shscramble control. ***P*<0.01.

SUPPLEMENTARY MATERIALS AND METHODS

Cell lysis, immunoprecipitation (IP), and western blotting

To make lysates for western blotting only, cells were washed once with ice-cold PBS, lysed with lysis buffer (Cell Signaling Technology, catalog number 9803). For IP of raptor, cells were lysed in ice-cold lysis buffer (40 mM HEPES, pH7.4, 120 mM NaCl, 10 mM sodium pyrophosphate, 50 mM NaF, 2 mM EDTA, 1x protease inhibitor cocktail (PIC)(Sigma-Aldrich, P8340), and 0.05% saponin) as previously described [1]. The lysate supernatant was collected after centrifugation at 10,000g for 10 min, and subjected to IP at 4 °C with raptor antibody and protein G Sepharose. Lysates and IP products were mixed with SDS sample buffer. All samples were boiled for 5 min and proteins were resolved on SDS-PAGE and transferred onto PVDF membranes (Millipore). Antibody incubations followed the manufacturers' recommendations. Horseradish peroxidase-conjugated secondary antibodies were detected with Chemiluminescent HRP Substrate (Millipore, P90720). Images were developed on x-ray film. Band intensities were quantified based on densitometry of the images using ImageJ software (NIH).

Oil Red O staining and lipid content assay

Oil Red O staining to measure lipid content was performed as previously reported[2] with minor modifications. Briefly, 3T3-L1 adipocytes were rinsed with PBS, fixed in 10% formalin for 1 hour, and stained with 0.35% (w/v) Oil Red O (ORO) solution in 60% (v/v) isopropanol for 10 min, followed by washing with water. Stained cells were examined under a laser-scanning microscope (Carl Zeiss, LSM 700) with a 10× objective lens, and bright-field images were captured using a LSM T-PMT camera (LSM 700, Carl Zeiss). The images were then processed in Photoshop CS5 (Adobe), where contrast and

brightness were adjusted. To determine lipid content, isopropanol (0.5 mL per well of a 12-well plate) was added to the stained cells to extract the dye, and the solution was transferred to a 96-well plate and measured using a spectrophotometer at 510 nm.

Quantitative RT-PCR

Total RNA was extracted from either undifferentiated or differentiating 3T3-L1 cells using Trizol Reagent (Thermo Fisher Scientific). cDNA was synthesized from 1 μg RNA using a TOPscript™ RT DryMIX kit (dT18 plus) (Enzynomics, RT200). Real-time PCR analysis was performed with a CFX384 C1000 Thermal Cycler (Bio-Rad) using TOPreal™ qPCR 2X PreMIX (SYBR Green with high ROX) (Enzynomics, RT501S). Mouse glyceraldehyde 3-phosphate dehydrogenase (GAPDH) was used to normalize gene expression and cyclophilin A was used as an internal control. A list of primer sequences is provided in Supplementary Table 1.

Supplementary Table 1. Primers used in the study

Gene	Sequence
PLD1 F	5' - AGTGCTTCAGACTTGTCCTGGGTT - 3'
PLD1 R	5' - TATGGTAGCGTTTCGAGCTGCTGT - 3'
PPARγ F	5' - ATCTTAACTGCCGGATCCAC- 3'
PPARγ R	5' - TGGTGATTTGTCCGTTGTCT- 3'
FAS F	5' - GCTGCGGAAACTTCAGGAAAT - 3'
FAS R	5' - AGAGACGTGTCACTCCTGGACTT - 3'
LPL F	5' - GGGAGTTTGGCTCCAGAGTTT -3'
LPL R	5' - TGTGTCTTCAGGGGTCCTTAG - 3'
C/EBPα F	5' - TGTTGGGGATTTGAGTCTGTG - 3'
C/EBPα R	5' - GGAAACCTGGCCTGTTGTAAG - 3'
aP2 F	5' - AAGGTGAAGAGCATCATAACCCT - 3'
aP2 R	5' - TCACGCCTTTCATAACACATTCC - 3'
Cyclophilin A F	5' - CAAGACTGAATGGCTGGATG - 3'
Cyclophilin A R	5' - TGGTGATCTTCTTGCTGGTC - 3'

SUPPLEMENTARY REFERENCES

1. Yoon, M.S. *et al.* Rapid Mitogenic Regulation of the mTORC1 Inhibitor, DEPTOR, by Phosphatidic Acid. *Mol Cell* **58**, 549-556 (2015).
2. Ramirez-Zacarias, J.L., Castro-Munozledo, F. & Kuri-Harcuch, W. Quantitation of adipose conversion and triglycerides by staining intracytoplasmic lipids with Oil red O. *Histochemistry* **97**, 493-497 (1992).
